# Curcumin Decreases Viability and Inhibits Proliferation of Imatinib-Sensitive and Imatinib-Resistant Chronic Myeloid Leukemia Cell Lines

**DOI:** 10.3390/metabo13010058

**Published:** 2022-12-30

**Authors:** Esma Bilajac, Lejla Mahmutović, Una Glamočlija, Amar Osmanović, Altijana Hromić-Jahjefendić, Murtaza M. Tambuwala, Mirza Suljagić

**Affiliations:** 1 Department of Genetics and Bioengineering, Faculty of Engineering and Natural Sciences, International University of Sarajevo, Hrasnička cesta 15, 71000 Sarajevo, Bosnia and Herzegovina; 2 Faculty of Pharmacy, University of Sarajevo, Zmaja od Bosne 8, 71000 Sarajevo, Bosnia and Herzegovina; 3 School of Medicine, University of Mostar, Zrinskog Frankopana 34, 88000 Mostar, Bosnia and Herzegovina; 4 Scientific-Research Unit, Bosnalijek JSC, Jukićeva 53, 71000 Sarajevo, Bosnia and Herzegovina; 5 Lincoln Medical School, University of Lincoln, Brayford Pool Campus, Lincoln LN6 7TS, UK; 6 3DBioLabs, FabLab B&H, University of Sarajevo Campus, Zmaja od Bosne 8, 71000 Sarajevo, Bosnia and Herzegovina

**Keywords:** curcumin, chronic myeloid leukemia, imatinib, imatinib resistance

## Abstract

Chronic myeloid leukemia (CML) is a myeloproliferative haematological malignancy characterized by constitutive activation of BCR-ABL1 tyrosine kinase in the majority of patients. BCR-ABL1 expression activates signaling pathways involved in cell proliferation and survival. Current treatment options for CML include tyrosine kinase inhibitors (TKI) with resistance as a major issue. Various treatment options for overcoming resistance are being investigated. Among them, phytochemical curcumin could play an important role. Curcumin has been found to exhibit anti-cancerous effects in various models, including CML, through regulation of multiple molecular signaling pathways contributing to tumorigenesis. We have evaluated curcumin’s effects on imatinib-sensitive LAMA84S and K562, as well as imatinib-resistant LAMA84R cell lines. Our results indicate a significant dose-dependent decrease in cell viability and proliferation of imatinib-sensitive and imatinib-resistant cell lines after curcumin treatment. Suppression of key signaling molecules regulating metabolic and proliferative events, such as Akt, P70S6K and NF-kB, was observed. Increased expression of caspase-3 suggests the potential pro-apoptotic effect of curcumin in the imatinib-resistant CML model. Additional in silico molecular docking studies revealed binding modes and affinities of curcumin with different targets and the results are in accordance with in vitro findings. Altogether, these results indicate the potential role of curcumin in the treatment of CML.

## 1. Introduction

Chronic myeloid leukemia (CML) is a myeloproliferative malignant disorder arising from the uncontrolled growth of hematopoietic myeloid lineage cells, accumulating in blood and bone marrow [1]. More than 95% of patients with CML are Philadelphia (Ph) positive with reciprocal translocation t(9;22)(q34;q11) generating BCR-ABL1 fusion protein with constitutive tyrosine kinase (TK) activity [2,3]. BCR-ABL1 oncoprotein activates a cascade of signaling pathways involved in cell proliferation, differentiation and apoptosis [3,4,5,6]. TKs are characterized with an ability to modify a wide range of cellular functions, while constitutive activity arising as a result of mutation or other factors leads to deregulations in cellular signaling cascades with a pathological outcome. Inhibition of TK activities with tyrosine kinase inhibitors (TKIs) can prevent the uncontrolled action of dysfunctional tyrosine kinases. TKIs disrupt the transduction of intracellular signaling of tyrosine kinases that phosphorylate substrate’s tyrosine residues. Depending on the type of the drug, TKIs bind to the ATP-binding site located in the catalytic region of inactive tyrosine kinase, blocking its function. Certain TKIs competitively bind the ATP-binding pocket of active TK or bind allosteric pockets either adjacent or far from ATP-binding sites. In addition, several TKIs possess a multiple TK binding modes. The antitumor mechanism of TKIs is observed through inhibition of cell division, repair machineries, induction of apoptosis and other. The development of first-generation TKIs for CML treatment, imatinib, has resulted in remarkable cytogenetic responses and a low probability of progression to the accelerated phase [7,8,9,10]. Imatinib, a non-ATP competitive inhibitor binds BCR-ABL kinase in its inactive conformation, specifically to the ATP binding site. This event prevents the transfer of crucial phosphate group to the protein substrate, thus blocking the activation of cell proliferation. The major problem arising following imatinib treatment is that more than 30% of treated patients develop imatinib resistance, with half of the patients expressing T315I point mutation in the BCR-ABL1 ATP-binding domain [3,11]. Consequently, recent advances in pharmacological research have resulted in the emergence of second- and third-generation TKIs in CML treatment, including dasatinib, bosutinib, and nilotinib with increased anti-leukemic activity and significant clinical outcome compared to imatinib treatment [3,11,12,13,14,15,16,17,18]. However, long-term toxicity and high cost of TKIs have been important concerns for the treatment of CML patients [3,18,19]. Alternative CML therapeutic options with high safety profiles and low prices are urgently needed.

Curcumin, a natural polyphenol derived from the rhizome of turmeric *Curcuma longa* exhibits anti-inflammatory, anti-oxidative, and anti-cancer properties in breast, lung, prostate, colon cancer, leukemia and other cancer types [20,21,22,23,24,25,26,27,28,29,30,31,32]. Curcumin inhibits cell proliferation, invasion, migration and induces cell cycle arrest and apoptosis of cancer cells [33,34]. Curcumin’s anti-tumor activity is observed through regulation of multiple intracellular signaling pathways, including the phosphatidylinositol 3 kinase/Akt (PI3K/Akt) signaling pathway, nuclear factor kappa B (NF-κB) signaling, mitogen activated protein kinase (MAPK) signaling, p53 signaling, apoptosis and many other events.

The PI3K pathway plays a critical role in the progression of cancer as a result of regulation of tumor cell growth, survival, proliferation, cell metabolism and metastasis.

The activation of PI3 kinase is stimulated by various extracellular signals that bind PI3K to the plasma membrane through scaffold proteins. Following stimulation, PI3K-p110α lipid substrate phosphatidylinositol-4,5-bisphosphate (PIP2) is transformed to phosphatidylinositol-3,4,5-triphosphate (PIP3). This event results in phosphorylation of serine/threonine kinase, Akt/protein kinase B (PKB) that promotes cell survival and growth. Loss of tumor suppressor phosphatase and tensin homolog (PTEN) that dephosphorylates PIP3 into PIP2 and mutations in *PIK3CA* gene that encodes p110α subunit are commonly observed in multiple cancer types.

Akt has an ability to singly regulate numerous processes, including activation of the NF-kB pathway that result in anti-apoptotic effects, as observed in lymphoma. PKB proto-oncogene promotes activation of mammalian target of rapamycin (mTOR) complex via inhibition of tumor suppressor, tuberous sclerosis 1/2 (TSC1/2), a guanosine triphosphatase Rheb-GTP-activating protein. Upon phosphorylation of TSC2 and PRAS40 as a part of mTORC1 complex, Akt stimulates mTORC1 activation and up-regulation that results in activity of 40S ribosomal protein S6 kinase 1 (S6K1) and eukaryotic initiation factor 4E binding protein (4EBP1), leading to protein translation and cellular growth. Up-regulation of mTOR results in synthesis of numerous proteins, including proteins implicated in cell cycle progression and others.

Moreover, the NF-κB signaling pathway has an important role in immune responses, inflammation, as well as cell proliferation, differentiation and survival. NF-kB represents a family of transcription factors (TFs), forming homodimers or heterodimers that bind to promoter regions of responsive genes, triggering cytokine and chemokine secretion. NF-kB family of TFs including RelA/p65, RelB/p50, c-Rel and others are found in its inactivated form in the cytoplasm, bound to inhibitory kappa B (IκB) protein.

NF-κB TFs are activated by different growth factors, cytokines, DNA damage, ionizing radiation, reactive oxygen species (ROS), and numerous other intracellular and/or extracellular stimuli. Through the canonical NF-κB pathway, these signals trigger the activation of IκB kinase (IKK) composed of three components, including IKKα, IKKβ and IKKγ. IKK phosphorylates IκB bound to NF-κB TFs, tagging it for degradation through E3 ubiquitin ligase-mediated proteasomal degradation machinery. This event results in translocation of NF-κB dimers into the nucleus and initiation of transcription of genes regulating numerous cellular functions.

NF-κB signaling is constitutively active in different cancer types. This activation results in up-regulation of anti-apoptotic genes that lead to prolonged cancer cell survival and proliferation. In addition, gene amplifications, point mutations and chromosomal truncations of NF-κB signaling genes are associated with occurrence of lymphoid malignancies and leukemia, underlining the direct oncogenic potential of NF-κB signaling.

**Figure 1:**
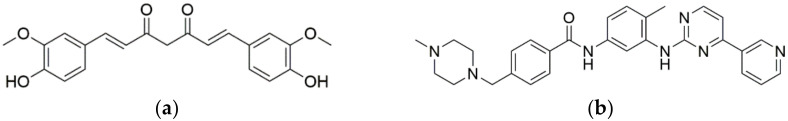
Chemical structures of curcumin (**a**) and imatinib (**b**). 2D structures of the curcumin and imatinib molecules were prepared using Spartan 14 software.

In leukemia cells, curcumin suppressed the phosphorylation and degradation of IκBα, therefore preventing nuclear translocation of NF-κB transcription factor to the nucleus. In other leukemia cell models, curcumin treatment resulted in decreased expression of signal transducer and activator of transcription 3 (STAT3), Akt, NF-κB targets, while inducing apoptosis through inhibition of X-linked inhibitor of apoptosis protein (XIAP) and overexpression of pro-apoptotic Bim protein. Moreover, it has been shown that curcumin improves the effects of imatinib treatment in sensitive and resistant CML cell lines [34,35]. In leukemic cells, curcumin is primarily taken up by mitochondria, collapses mitochondrial potential and induces the permeability pore formation via mitochondrial Ca^2+^ overload. Chemical structures of curcumin and imatinib are presented in Figure 1a,b.

However, data showing the mechanism of action of curcumin/imatinib co-treatment and description of molecular events in imatinib resistant clones still need to be revealed.

Therefore, in this study we aimed to evaluate curcumin’s effects on cell viability, proliferation and activity of key molecular signaling pathways in imatinib-sensitive (LAMA84S and K562) and imatinib-resistant (LAMA84R) cell lines. These targets represent key molecules in promoting growth, proliferation and survival of leukemia cells, including p-Akt, its’ downstream target p-P70S6K, and p-NF-κB protein. In addition, we have also evaluated the effect of curcumin on the expression of caspase-3 as an important hallmark of cell apoptosis. The potential antitumor effects of curcumin in leukemic cells sensitive and resistant to imatinib therapy were evaluated in order to understand possible therapeutic action of curcumin in these cell models, especially in cells characterized with imatinib resistance. Such an approach represents an important step for future perspectives where curcumin co-treatment of imatinib-resistant clones would potentially increase the therapeutic response where current mono-treatment options fail to induce the desired outcome.

## 2. Materials and Methods

### 2.1. Cell Lines and Cultures

Imatinib-sensitive LAMA84S and imatinib-resistant LAMA84R cell lines were kindly provided by Dr. Aleksandar Radujković (University of Heidelberg, Heidelberg, Germany). K562 cell line was a generous gift from Dr. Ivana Novak (University of Split, Split, Croatia). Cells were grown at 37 °C and 5% CO_2_ in RPMI-1640 medium, supplemented with 10% Fetal Bovine Serum (FBS), 100 U/mL penicillin, 100 μ/mL streptomycin, 10 mM HEPES, 1 mM sodium pyruvate and 1% of a non-essential amino acid (Sigma-Aldrich, Gillingham, UK). Additionally, the LAMA84R cell line was cultured in RPMI-1640 containing 1 μM imatinib (Sigma-Aldrich, Gillingham, UK). Curcumin (Merck Millipore, Darmstadt, Germany) was dissolved in DMSO (Sigma-Aldrich, Gillingham, UK) to obtain a 10 mM concentration and further diluted in 1X phosphate-buffered saline (PBS) (Fisher Bioreagents, Waltham, MA, USA) to 1 mM concentration. Fresh curcumin solution was prepared for each experiment.

### 2.2. Cell Viability Assay

Cell viability was determined by WST-8 assay (Bimake, Houston, TX, USA). Cells were plated in triplicates in a 96-well plate at an optimum seeding density of 2.5 × 10^4^ cells/well. Cells were treated with a range of curcumin concentrations adopted from the literature data, while 0.1% DMSO was used as negative control. Imatinib-sensitive cells were treated with 10–30 μM curcumin, while imatinib-resistant cells were treated with 25–200 μM curcumin concentrations. Cell viability experiments were performed at 48 h period. After incubation with the treatment, 10 μL of CCK-8 was added in each well and the plates were incubated at 37 °C for 2 h. The absorbance values were measured at 450 nm and the reference wavelength of 620 nm in a Multiscan FC microplate reader (Thermo Fisher Scientific, Waltham, MA, USA). The half-maximal inhibitory concentration 50 (IC50) was calculated based on the dose-response curve. Cell death was determined by trypan blue exclusion assay. Cell lines were treated with different curcumin concentrations indicated above for 24, 48, and 72 h. Cells were mixed and stained with 0.4% filtered trypan blue solution (Gibco^TM^ Life Technologies, Waltham, MA, USA) and counted with Countess II FL Automated Cell Counter (Thermo Fisher Scientific, Waltham, MA, USA). The results are represented as a percentage relative to the negative control (0.1% DMSO) set as 100% of cell viability.

### 2.3. Cell Proliferation Assay

K562 and LAMA84R cell lines were seeded in triplicate in a 96-well plate at a seeding density of 2.5 × 10^4^ cells/well and treated for 48 h, where 0.1% DMSO was used as negative control. Cell proliferation rates were evaluated by Cell Proliferation ELISA, BrdU colorimetric assay kit (Roche Applied Science, Basel, Switzerland), according to the manufacturer’s protocol (version 16).

### 2.4. Molecular Docking Study

The molecular docking study was set up in YASARA Structure 19.12.14 software [36,37] and carried out using AutoDock version 4.2 [38]. The crystal structures of I-Kappa-B-Alpha/NF-Kappa-B (PDB ID: 1NFI) [39], Akt1 (PDB ID: 4EKK) [40] and NF-kappa-B inducing kinase (PDB IDs: 4G3D, 4G3E, 4G3F and 4G3G) [41], protein kinase B unphosphorylated (PDB ID: 1GZO) [42], and phosphorylated p70S6K1 (PDB ID: 3A62) [43] were downloaded from Protein Data Bank and used as a target molecules. The protein structures were prepared by removing water molecules, adding polar hydrogen atoms and optimizing in the AMBER03 force field [44]. The 3D structures of the curcumin and imatinib molecules were prepared and geometries optimized by the density functional theory (DFT) (B3LYP/6-31G* basis set) using Spartan 14 software [45]. Molecular docking analyses were performed using either the blind docking method (searching the whole protein for potential binding sites) or setting up the search area box around a specific binding pocket. The Lamarckian genetic algorithm was employed with the following parameters: 150 docking runs with a maximum of 15,000,000 energy evaluations and 27,000 generations for each run, with a grid point spacing of 0.375 Å, providing this way the lowest energy docked structures. For the comparison and analyses of obtained protein-ligand complexes, a multiple structural alignment algorithm MUSTANG was used [46].

### 2.5. Western Blot Analysis

For Western blot analysis, cells were seeded in a 6-well plate at a seeding density of 1 × 10^6^ cells/well and treated with curcumin concentrations for 48 h. Cells treated with 0.1% DMSO were used as a negative control. Cells were lysed in ice-cold RIPA buffer supplemented with protease and phosphatase inhibitor cocktails (Sigma-Aldrich, Gillingham, UK). All samples were separated by 12% SDS-polyacrylamide gel electrophoresis (SDS-PAGE) and transferred onto a polyvinylidene difluoride (PVDF) nitrocellulose membrane (Merck Millipore, Darmstadt, Germany). The membrane was blocked with 5% BSA in Tris-buffered saline supplemented with 0.1% Tween-20 buffer (Sigma-Aldrich, Gillingham, UK). Western blot analyses were performed using primary antibodies against p-NF-κB (#3033), p-P70S6K (#9204), cleaved caspase-3 (#9661), p-Akt (#4060) and β-actin (#3700) (Cell Signaling Technology, Danvers, MA, USA). The signals were detected using Amersham^TM^ ECL^TM^ Prime Western Blotting Detection Reagent (GE Healthcare Life Sciences, Amersham, UK). Protein bands were visualized by Molecular Imager ChemiDoc^TM^ XRS+ Imaging System (Bio-Rad, Hercules, CA, USA) and analyzed by Image Lab software (version 6.0) (Bio-Rad, Hercules, CA, USA).

### 2.6. Statistical Analysis

The results of different experiments were represented as means ± standard deviation (SD). The differences between curcumin-treated cells and the negative control group were analyzed using GraphPad Prism software (version 8.3, Prism, San Diego, CA, USA). *t*-test was used for comparison of treatment and control in BrdU assay. One-way ANOVA Dunnett’s multiple comparison analysis was used for statistical evaluation. The normal distribution of variances was tested by the D’Agostino-Pearson normality test. Shapiro–Wilk test was used where normality test indicated normal distribution of variances (*p* > 0.05), whereas Kolmogorov–Smirnov test was used otherwise. *p* < 0.05 was considered as level of statistical significance with the next levels presented through the text: * *p* < 0.05, ** *p* < 0.01, *** *p* < 0.001, **** *p* < 0.0001.

## 3. Results

### 3.1. Curcumin Induced Dose-Dependent Decrease of Cell Viability of Imatinib-Sensitive and Imatinib-Resistant Cell Lines

**Figure 2:**
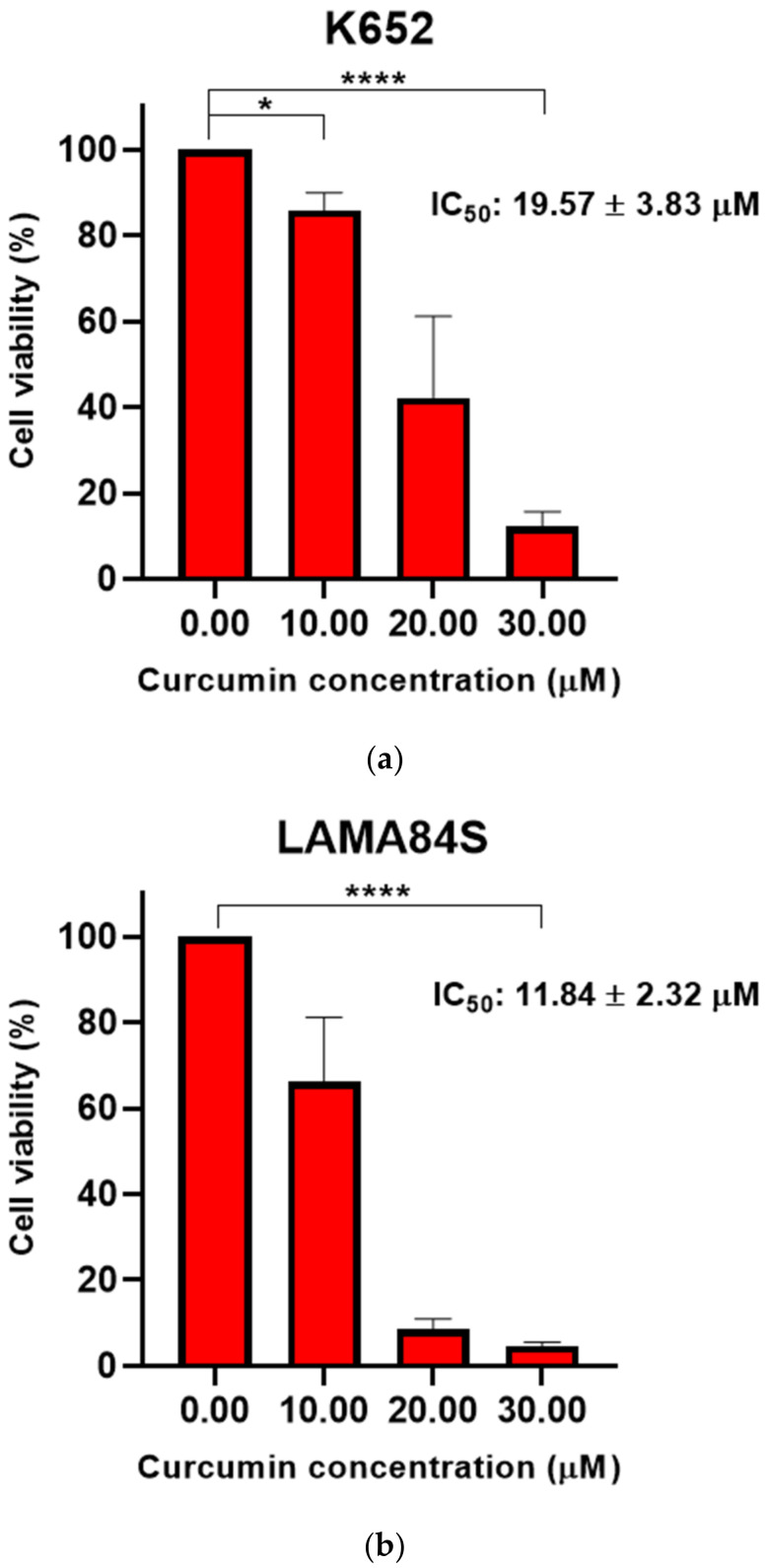

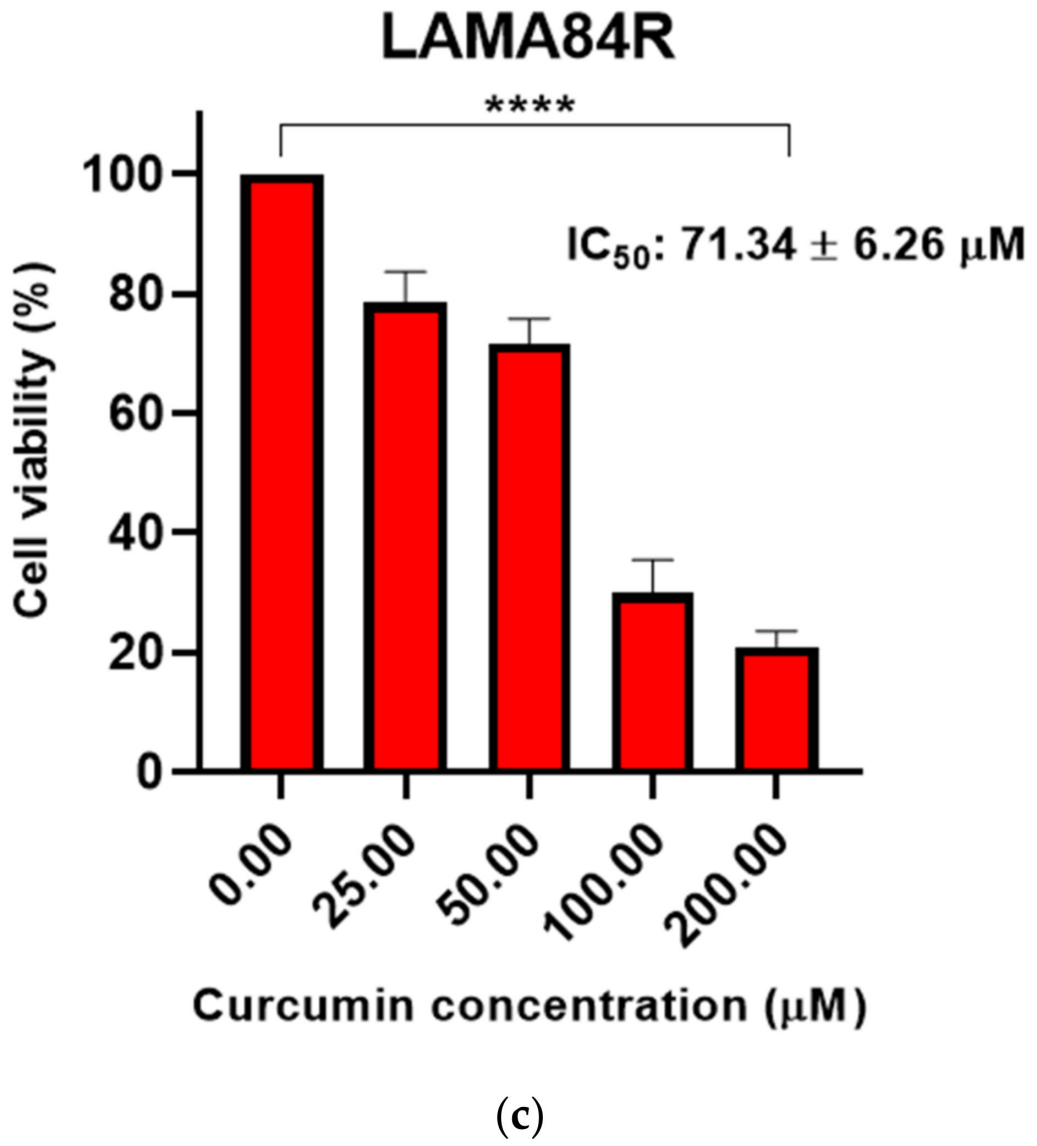
Dose-dependent effects of curcumin on cell viability of (**a**) K562, (**b**) LAMA84S, and (**c**) LAMA84R cell lines measured by WST-8 assay after 48 h treatment. The mean ± standard deviations are shown. The results were obtained from the three independent experiments. * *p* < 0.05, **** *p* < 0.0001.

Imatinib-sensitive LAMA84S and K562 cell lines were treated with a range of curcumin concentrations (10–30 μM) for 48 h. The results indicate a significant dose-dependent decrease in cell viability at different treatment concentrations compared to the negative control (0.1% DMSO) (Figure 2a–c).

LAMA84S cell line showed greater sensitivity to curcumin than K562 cells. Curcumin (25–200 μM) evinced a significant dose-dependent decrease of viability in imatinib-resistant LAMA84R cells after 48 h treatment (Figure 2c).

**Figure 3:**
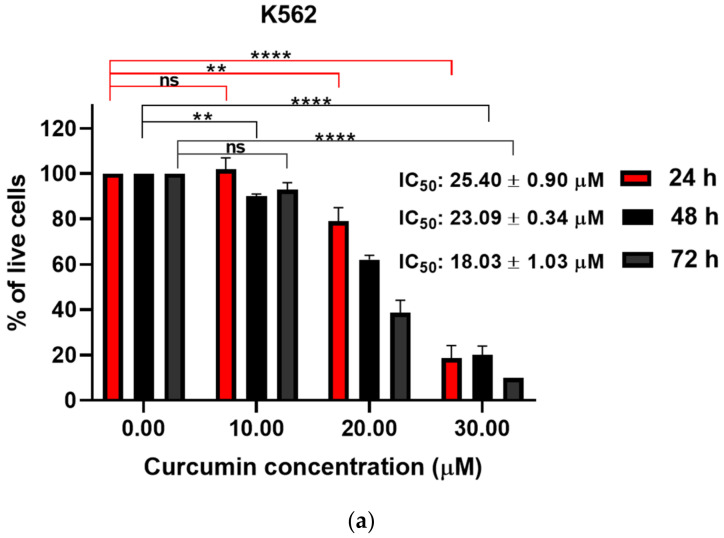

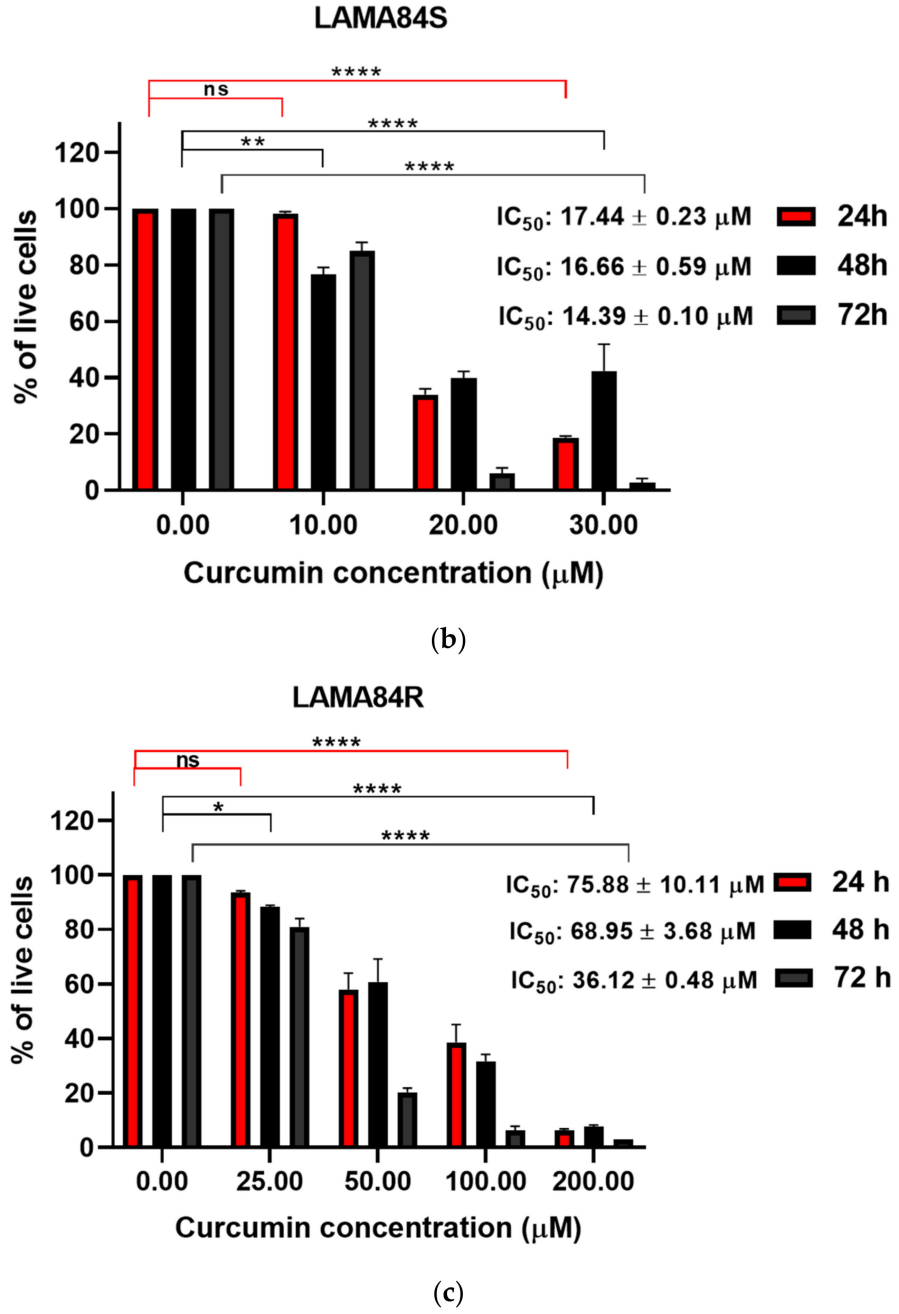
Time- and dose-dependent effects of curcumin on cell death of K562 (**a**), LAMA84S (**b**), and LAMA84R (**c**) cell lines evaluated through trypan blue exclusion assay. The mean ± standard deviations are shown. The results were obtained from at least three independent experiments. ns – not significant, * *p* < 0.05, ** *p* < 0.005, **** *p* < 0.0001.

We have performed the trypan blue exclusion assay to assess cell death of imatinib-sensitive and imatinib-resistant cell lines. A significant, dose-dependent increase in percentage of dead cells was observed in all cell lines treated with curcumin (Figure 3a–c).

The comparison of curcumin IC_50_ values is summarized in Table 1.

### 3.2. Curcumin Induced Dose-Dependent Decrease of Cell Proliferation of Imatinib-Sensitive and Imatinib-Resistant Cell Lines

We have performed a BrdU incorporation assay to evaluate the effects of curcumin on the proliferation rate of K562 and LAMA84R cell lines. For this analysis, K562 cells were treated with 20 µM while LAMA84R cells were treated with 70 µM curcumin (based on IC_50_ values determined by WST-8 assay for 48 h).

**Figure 4:**
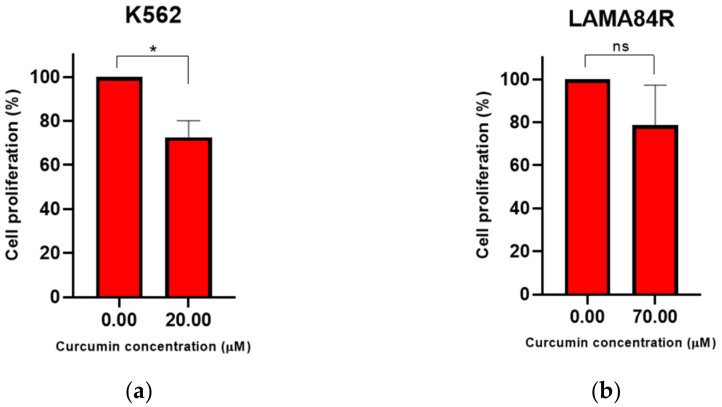
Curcumin effects on K562 (**a**) and LAMA84R (**b**) cell proliferation rates evaluated through BrdU incorporation assay after 48 h of curcumin treatment. The mean ± standard deviations are shown. ns – not significant, * *p* < 0.05.

The proliferation rate of K562 cells decreased significantly after incubation with curcumin (Figure 4a,b).

### 3.3. Molecular Docking Study of Curcumin and Imatinib with Protein Targets

A molecular docking study on curcumin and imatinib with target proteins revealed interesting insight into binding modes and affinities (Table 2). Predicted values indicate that curcumin forms the most significant complexes with non-phosphorylated protein kinase B and Akt1, followed by complexes with I-Kappa-B-Alpha/NF-Kappa-B and NF-kappaB inducing kinase. These complexes were characterized by binding energy values lower than −10 kcal/mol and dissociation constants lower than 0.04 μM.

Besides hydrogen bonds (H-bonds) in curcumin’s and imatinib’s binding to target proteins, other types of bonds were also involved, such as hydrophobic, π–π and cation-π, which were not shown for clarity, but are given in Supplementary Materials Figures S1–S7.

### 3.4. Analysis of Protein Targets of Curcumin in K562 and LAMA84R Cell Lines

Western blot analysis was performed to evaluate the effects of curcumin on expression levels of proteins involved in different molecular signaling pathways regulating metabolic activity, proliferation and apoptosis in K562 and LAMA84R cell lines. Cells were treated with different curcumin concentrations for 48 h.

**Figure 5:**
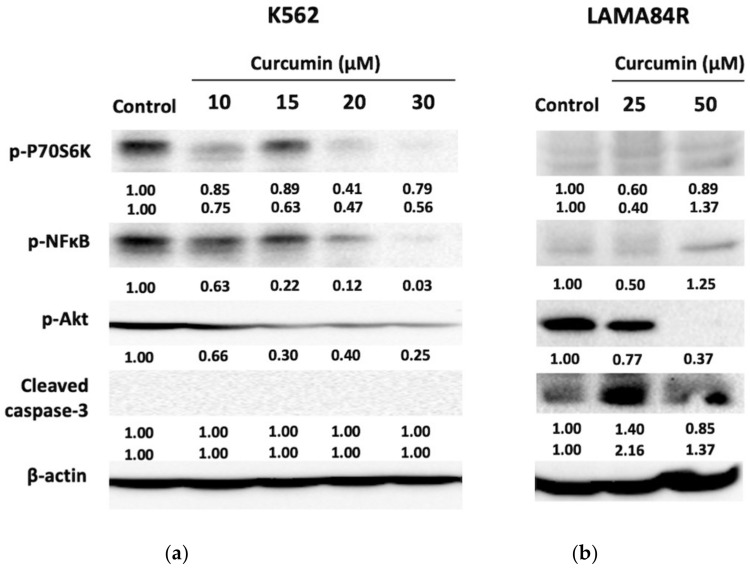
Analysis of protein expression/activity upon 48 h curcumin treatment of K562 (**a**) and LAMA84R (**b**) cell lines. Numbers below the bands represent a net image fold difference in the band intensity compared to a control and normalized against beta-actin.

In the K562 cell line we observed dose-dependent inhibition of transcription factor p-NF-κB activity after curcumin treatment (Figure 5a). At the highest curcumin concentration, phosphorylated levels of NF-κB have decreased by 97% as indicated by densitometry analysis. Moreover, curcumin treatment induced dose-dependent suppression of p-Akt activity, together with its downstream target p-P70S6K, suggesting its mechanism of action in imatinib-sensitive cells.

In LAMA84R imatinib-resistant cells, curcumin treatment resulted in a dose-dependent decrease of p-Akt levels, with a dramatic effect on its expression at a higher curcumin concentration (50 μM) (Figure 5b). The minimum concentration of curcumin used in our study for LAMA84R cell line (25 μM) decreased phosphorylation of NF-κB by 50% when compared to negative control. Interestingly, compared to negative control, increased curcumin concentration (50 μM) stimulated the expression of p-NF-κB, responsible for cell proliferation and differentiation. Similar effects of curcumin in the LAMA84R cell line were observed with the expression of p-P70S6K. In this case, lower curcumin concentration down-regulated the p-p70S6K expression involved in cell growth and G1 cell cycle progression, while the same effect was not observed at twice higher curcumin concentration that stimulated the phosphorylation of the mentioned protein kinase.

Additionally, we have evaluated the expression levels of caspase-3 protein included in the final step of programmed cell death. In K562 cells, the caspase-3 expression could not be detected. Treatment of imatinib-resistant cells with both curcumin concentrations stimulated the expression of caspase-3 compared to the control.

In summary, our results suggest promising antitumor role of curcumin and its’ ability to regulate the activity of main protein targets of PI3K/Akt and NF-κB signaling in imatinib-sensitive and imatinib-resistant cell lines.

## 4. Discussion

In our study, curcumin has shown dose-dependent effects on cell viability, death and proliferation in CML cells sensitive and resistant to imatinib. Curcumin is a very lipophilic molecule which is rapidly taken up by the cells, accumulating at the ER, lysosomes, and near mitochondria, without internal mitochondrial accumulation. Intracellular localization of curcumin in imatinib-sensitive and imatinib-resistant cells remains to be evaluated in the future. Moreover, numerous reports suggest selective cytotoxicity of curcumin in cancer cell lines, with its’ limited cytotoxic effect in healthy in vitro cell models. Pignanelli et al. (2017) showed that curcumin and its analogues selectively induced apoptosis in several cancer cell lines, without inducing apoptosis in normal-derived colon mucosa (NCM460) cells, non-tumorigenic epithelial breast cancer cells (MCF10A) and peripheral blood mononuclear cells (PBMC) obtained from healthy donors. Similarly, other reports have shown that curcumin induces apoptosis in cutaneous T-cell lymphoma cell lines, with more prominent apoptotic activity on PBMC obtained from a patient in comparison to healthy control groups. Other reports also show that curcumin induces apoptosis in B-cell chronic lymphocytic leukemia with four-fold lower effective concentration 50 (EC_50_) in cancer cells compared to normal mononuclear cells. One of the potential reasons for curcumin’s selectivity toward cancer cells can be explained by its potential to increase the cellular reactive oxygen species (ROS) levels. Phytochemicals with known safety profile isolated from natural sources, such as curcumin, can be used in combinatorial treatments with other drugs. This approach allows application of different substances targeting diverse signaling pathways to increase therapeutic effectiveness, decrease adverse effects and overcome cancer resistance to the therapy. Drug repurposing is one of the important admissions for combinatorial treatment development. Using data from clinical studies and practice, drugs with well-known safety profile approved for different conditions represent an opportunity for development of new drug combinations that target different members of intracellular signaling pathways deregulated in cancer. Such an approach is used in CML models [47,48,49,50]. Several researches propose the importance of curcumin as adjuvant therapy in CML with its ability to overcome the imatinib resistance in in vitro cell models [33,51,52]. As indicated by WST-8 assay, our data demonstrate a significant dose-depended decrease in cell viability upon curcumin treatment of imatinib-sensitive and imatinib-resistant cell lines.

WST-8 assay is a highly sensitive, non-toxic colorimetric assay. Cell Counting Kit-8 (CCK-8) uses highly water-soluble tetrazolium salt WST-8 [2-(2-methoxy-4-nitrophenyl)-3-(4-nitrophenyl)-5-(2,4 disulfophenyl)-2*H*-tetrazolium, monosodium salt] that is reduced by the activity of mitochondrial NAD(P)H enzymes to produce formazan orange dye by bio-reduction process in the presence of specific electron carrier 1-methoxy-5-methylphenazinium methyl sulfate (1-methoxy PMS). The amount of formazan dye produced in the reaction is directly proportional to the number of living cells. In line with the study done by Mutlu Altundağ et al. (2018), our results have shown a significant decrease in K562 cell viability upon 48 h treatment [51]. The viability of imatinib-resistant cell lines has also decreased significantly upon increased curcumin concentrations (100 and 200 μM), while no significant effect on cell viability was observed with low compound concentrations used for the treatment of imatinib-sensitive cell line at three different incubation periods. Imatinib-resistant leukemic clones exhibit various mechanisms of drug resistance, including over-expression and point mutations in the BCR-ABL1 TK domain, as well as increased expression of P-glycoprotein (MDR-1) responsible for drug resistance [53,54]. LAMA84R cell line used in this study is characterized with an increased expression of BCR-ABL and P-glycoprotein as reported by Radujković and colleagues. In our study, the lower sensitivity of resistant cells toward curcumin treatment might occur as a consequence of BCR-ABL and MDR-1 expression. We can surmise that the inhibition of MDR-1 would increase the sensitivity of resistant cells toward curcumin treatment. Furthermore, we can assume that this approach would significantly affect the cellular sensitivity to the drug, however, this remains to be evaluated in the future. In addition, for the future perspectives, intracellular localization of curcumin in imatinib-sensitive and imatinib-resistant cells should be evaluated using confocal microscopy in order to understand compound’s accumulation inside both cell models. Although specific interests are focused on overcoming the drug resistance by curcumin due to the low compound toxicity [54], still low bioavailability and solubility of curcumin represent a great challenge in curcumin treatment. Former studies suggested the design of curcumin analogues and derivatives to prevent drug resistance and improve compound bioavailability [53]. Accordingly, curcumin derivative C817 has been shown to inhibit proliferation and down-regulate the BCR-ABL1 gene expression in imatinib-resistant CML cell models [54]. Another curcumin derivatives, “Compound 10”, C086, and C1206 have shown more significant anti-tumor properties compared to parent compound treatment [53,54,55]. Even though we did not observe a significant effect of curcumin on the viability of imatinib-resistant cells at lower concentrations, higher curcumin concentration triggered a significant decrease in cell viability of imatinib-resistant cells at 48 h. Compared to the negative control group, our results indicate a decrease in LAMA84R cell viability for 70% and 80% at curcumin concentrations of 100 and 200 μM, respectively. One of the possible reasons for an insignificant change in cell viability upon curcumin treatment of resistant cells at low curcumin concentration might be poor compound bioavailability and other mechanisms correlated to drug resistance since a similar scenario was observed in other cancer models [56,57,58]. However, importance of curcumin in overcoming the imatinib resistance in CML models is mainly achieved by the use of various newly synthesized curcumin analogues and derivatives, as mentioned previously [53,54,55,59].

Curcumin’s effect on the cell death of CML cell lines was evaluated by a simple, low-cost and rapid trypan blue exclusion assay. Trypan blue dye is used for direct determination of the number of viable cells present in a suspension, where live cells are characterized with intact or impermeable cell membrane that excludes certain dye(s), such as trypan blue. Reversely, the dye enters dead cells with compromised cell membranes and binds to intracellular proteins. As a result, viable cells retain clear cytoplasm, where dead, non-viable cells are stained, appearing with blue-colored cytoplasm. The results indicated dose- and time-dependent increase in the percentage of dead cell following curcumin treatment. We have evaluated the effects of curcumin on the proliferation rate by BrdU incorporation assay. In this assay, 5-bromo-2′-deoxyuridine (BrdU), representing a thymidine analogue is incorporated into newly synthesized DNA during cell proliferation. Following DNA denaturation, anti-BrdU antibody linked to a dye is applied to bind BrdU label and the quantity of a reaction product is measured by spectrophotometer. The absorbance values obtained from BrdU immunoassay are directly proportional to the amount of newly synthesized DNA and a number of proliferating cells.

Our results suggest significant decrease of the cell proliferation rate of imatinib-sensitive and a trend in decrease of proliferation rate in imatinib-resistant cell lines upon curcumin treatment. Contrary to our study, Wolanin (2006) did not show a significant decrease in cell proliferation rate upon 20 μM curcumin treatment of imatinib-sensitive CML cell models by BrdU incorporation assay. However, in the same study, curcumin has been shown to regulate proliferation in CML cell models through down-regulation of protein expression, including Cyclin D2 [59].

To understand the binding affinity of curcumin to several key signaling molecules contributing to tumorigenesis, we performed molecular docking analysis. Molecular docking study of curcumin and its derivatives was so far reported by different research groups. Using computational approach, Eryanti et al. (2017) evaluated the activity of eight different curcumin analogues against human promyelocytic leukemia cell line (HL-60). Docking and molecular dynamic simulation data revealed great binding affinities of curcumin analogues toward telomerase reverse transcriptase (TERT) (PDB ID: 3DU6), a catalytic telomerase subunit with an important role in maintaining chromosome stability. Abnormal telomerase activity has been linked to cancer development, including leukemia [60,61]. Molecular docking analysis suggests that presence of hydroxyl group in meta position could improve the biological activity of curcumin derivatives [60]. Similarly, molecular binding modes of curcumin derivatives to human topoisomerase I and II, enzymes important for management of DNA topology during different cellular processes was also reported through literature data. The results showed that curcumin derivatives are able to dock at the DNA cleavage site, similar mode as observed with topoisomerase I/II inhibitors. The results indicated that cyclocurcumin and curcumin sulphate derivatives employed lowest binding energies, representing these derivatives as potential dual topoisomerase I/II inhibitors [62]. In our study, we compared the binding affinities of curcumin (parental structure) and imatinib as a first generation TKI toward several targets involved in tumorigenesis.

Analyzing the interaction between I-Kappa-B-Alpha/NF-Kappa-B (PDB ID: 1NFI) and curcumin, molecular docking revealed strong binding affinity and stability parameters of the formed complexes. Lower values of the binding energies indicate better stability of the formed complexes between curcumin/imatinib and target protein. Additionally, the values of dissociation constants were also addressed. This parameter indicates the concentration of a ligand required to bind half of the target molecules.

Curcumin bound to I-Kappa-B-Alpha/NF-Kappa-B (Figure S1, Supplementary Materials) with slightly lower affinity compared to imatinib, however, curcumin occupied the same binding spot as imatinib during the blind docking analysis, which supports other obtained docking parameters (Figure S2, Supplementary Materials).

A similar case of overlapping of curcumin and imatinib was observed in blind docking analysis of NF-kappaB inducing kinase (PDB ID: 4G3D). Although curcumin and imatinib overlapped here only partially (Figure S3, Supplementary Materials) compared to I-Kappa-B-Alpha/NF-Kappa-B, it is of great importance to point out the similar affinity of both molecules for this target. According to obtained docking parameters, curcumin bound NF-kappaB inducing kinase with a greater affinity than imatinib (Table 2). Dissociation constant values indicated that Curcumin concentration required to occupy 50% of the target protein molecule is 15 times lower than the concentration of imatinib.

Molecular docking analysis showed overall best binding prediction for curcumin with unphosphorylated protein kinase B (PDB ID: 1GZO). Overlapping of curcumin and imatinib molecules was here also partial (Figure S4, Supplementary Materials), with Gly 335-Trp 334 as important site for hydrogen bond formation and stabilization of the complex.

On the other side, docking analyses focused on specific binding pockets within the target proteins showed that curcumin formed more stable complexes than imatinib with all tested targets. The binding pocket for Akt1 was defined by removing the adenylyl imidodiphosphate (AMP-PNP) molecule from the PDB ID: 4EKK complex. Here, curcumin was effective at an almost 13 times lower concentration than imatinib at which half the protein molecules would have a ligand bound. Asp 439 is important here in forming the stable complex with this target for both ligands (Figure S5, Supplementary Materials).

For defining the binding pocket within human NF-kappa B inducing kinase, similar murine protein complexes with inhibitors were used as models. Three crystal structures of murine NF-kappaB inducing kinase bound to different ligands (PDB IDs: 4G3E, 4G3F and 4G3G) were aligned, together with human NF-kappaB inducing kinase (PDB ID: 4G3D), and space with overlapping ligands was defined as the binding pocket. According to this analysis, curcumin again showed significant binding affinity (Figure S6, Supplementary Materials), while imatinib showed a poor binding affinity with an extremely high dissociation constant (Table 2).

Phosphorylated p70S6K1 (PDB ID: 3A62) binding pocket was easily defined by removing present ligands in the complex. Even though curcumin binding affinities to phosphorylated p70S6K1 target is to certain extent weaker compared to previous targets (Figure S7, Supplementary Materials), however, it is important to mention that the predicted binding parameters are still favorable (Table 2), indicating stable complexes with observed H-bonds.

To the best of our knowledge, this is the first report comparing binding affinities of curcumin and imatinib toward several targets involved in cancerogenesis using molecular docking approach. Even though literature data suggest lower binding energies of curcumin derivatives to certain targets, still it is important to emphasize that our data suggest great binding affinities of parental curcumin compound. In silico testing is an important approach in preclinical drug design. Molecular docking software is used to determine ligand affinity toward certain target or macromolecule by using accurate structural models of the target. However, the candidate compounds with promising activities through in silico approach still need to be validated for potential pharmaceutical activity in in vitro settings. Complex environment, changes in pH values, activation of molecular signaling responses and drug bioavailability are only some of the important concerns that can affect in vitro results and drug activity. Therefore, activities of drug candidates have to be validated in in vitro settings since in silico approach offers an insight into binding modes of a drug toward certain targets, however, in virtual settings. The predicted dissociation constants of curcumin toward certain targets in this research is in a nanomolar range, while in vitro assays show antitumor activities of curcumin in micromolar values. Besides factors that affect drug activity mentioned above, curcumin’s low bioavailability has for long been a focus of concern. Several factors influencing low plasma and tissue levels of curcumin include rapid metabolism and systemic elimination, and initially poor absorption. Several approaches suggest the use of adjuvants or packing of curcumin into nanoparticles, liposomes, phospholipid complexes or use of curcumin derivatives as explained above. Molecular docking analysis was an important step in selection of targets for Western blot analysis.

Our results indicate that curcumin regulates the activity of different molecular targets involved in cell proliferation and apoptosis in CML cells sensitive (K562) and resistant (LAMA84R) to imatinib therapy. This is in accordance to previous studies in different cancer models [32,34,63]. Curcumin has been shown to regulate the expression levels of various molecular targets involved in different signaling pathways, including signaling pathways regulating apoptosis, cell survival and cell differentiation [35,52,64]. In line with other studies [34], our data have shown a dose-dependent decrease of p-Akt in both, imatinib-sensitive and imatinib-resistant cell lines, with a dramatic decrease of phosphorylated Akt in the LAMA84R cell line at 50 μM curcumin treatment. These results point out the effect of curcumin in the down-regulation of one of the key proteins involved in the PI3K/Akt signaling pathway in both cell lines.

Cleaved caspase-3 is associated with the mitochondrial apoptotic pathway and various studies have reported caspase-3 cleavage in different cancer models upon curcumin treatment [53]. In this study, we did not observe the expression of caspase-3 in the imatinib-sensitive CML model. One of the possible reasons might be the prolonged curcumin incubation period used in our experiments (treatment of 48 h). According to the related studies, expression of caspase-3 upon curcumin treatment in chronic myeloid cells is observed in a time frame of 3–24 h [65,66], and to our knowledge, there are no published data on the curcumin-stimulated expression of caspase-3 upon a treatment period of 48 h. In addition, the expression of caspase-3 upon 30 μM curcumin treatment of the K562 cell line has been observed after 12 h of treatment, with decreased expression after incubation for 24 h [65]. Thus, undetectable expression of caspase-3 upon 48 h is not surprising. However, in our experiment, expression of caspase-3 was dose-dependently induced in the imatinib-resistant model after a period of 48 h. Although activation of stream protein caspases, including caspase-3 has been observed upon treatment with curcumin derivatives and analogues in imatinib-resistant CML cells [54,55] at a lower incubation period, still, curcumin derivatives represent chemically different structures from the parent compound. Altogether, these data point out different curcumin effects dependent on cell type and research model, including imatinib-sensitive and imatinib-resistant cell lines. Taking together these findings, other proteins involved in the regulation of apoptosis, upon curcumin treatment need to be evaluated in the future.

In correlation with Reuter et al. (2009), our results indicate down-regulation of p-NF-κB at 30 μM curcumin treatment in the imatinib-sensitive cell line [64]. Moreover, in K562 cells, curcumin has also induced dose-dependent inhibition of p-P70S6K required for cell survival and growth. These data underline the effects of curcumin in suppressing the activity of proteins involved in cell proliferation, differentiation and cell cycle.

Moreover, decreased levels of phosphorylated Akt were seen in imatinib-resistant cell clones at higher curcumin concentration, together with the induction of cleaved caspase-3 levels. Characterized with drug-resistance cell mechanisms to escape cell death and promote cell proliferation [53], other potential mechanisms of curcumin effect in the LAMA84R cell line need to be considered, including adaptive cell response linked to curcumin in previously reported results [67].

In summary, our results indicate a significant dose-dependent effect of curcumin on cell viability of imatinib-sensitive and imatinib-resistant CML cell lines. These data indicate the inhibitory capacity of curcumin on the proliferation of K562 and LAMA84R cell lines. Western blot analysis has shown dose-dependent suppression of phosphorylated Akt, P70S6K, and NF-κB, protein levels in the K562 cell line. Additionally, curcumin treatment of imatinib-resistant cells has induced the expression of caspase-3 as one of the final hallmarks of apoptosis. Taking together the current analysis, it is important to highlight the potential therapeutic role of curcumin in both TKI-sensitive and -resistant CML cell models.

## 5. Conclusions

**Figure 6:**
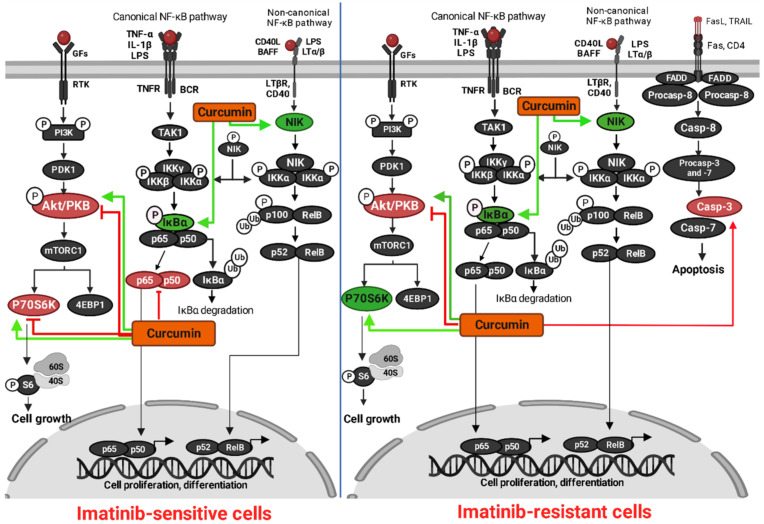
Mechanism of action of curcumin in imatinib-sensitive and imatinib-resistant CML cells evaluated through in vitro (red) and in silico (green) analysis. Shown by Western blot analysis, curcumin decreased the expression levels of phosphorylated p-NF-κB, p-Akt and p-P70S6K in imatinib-sensitive cells (red). In imatinib-resistant cells, curcumin induced decreased expression of phosphorylated p-Akt, with over-expression of cleaved caspase-3 as an important hallmark of apoptosis. The non-arrow/inhibitory lines show inhibition of the targets by curcumin In silico molecular docking analysis showed significant binding affinities of curcumin toward main players involved in promotion of cancer cell growth, proliferation and differentiation (green). The figure was created by BioRender.com (accessed on 20 October 2022).

The results of our study indicate a significant and dose-dependent decrease of cell viability of imatinib-sensitive and imatinib-resistant cell lines after curcumin treatment, with a trend in decrease of percentage of proliferation rate in both leukemia models. Molecular docking analysis revealed great binding affinities of curcumin to several molecular targets involved in tumorigenesis, with similar or higher affinity compared to imatinib, the first generation TKI used for CML treatment. We have shown that curcumin treatment provokes the over-expression of caspase-3 in resistant clones, as one of the final hallmarks of the mitochondrial apoptotic pathway. In this research, curcumin induced dose-dependent phosphorylation inhibition of Akt protein, as one of the major signaling molecules in the PI3K/Akt/mTOR signaling pathway. Finally, the summary of our results points out the possible therapeutic effect of curcumin in imatinib-resistant cell lines through regulation of major proteins of the intrinsic apoptotic pathway, as well as cell proliferation and viability. The graphical representation of our findings is shown in Figure 6.

## Figures and Tables

**Table 1:** IC_50_ of K562, LAMA84S, and LAMA84R cell lines upon curcumin treatment evaluated through WST-8 cell viability and trypan blue exclusion assays at 48 h.

IC_50_						
Cell Line	K562		LAMA84S		LAMA84R	
Assay (48 h)	WST-8	Trypan blue	WST-8	Trypan blue	WST-8	Trypan blue
	20.11 ± 3.10	21.62 ± 0.37	12.60 ± 2.16	16.66 ± 0.59	71.34 ± 6.26	68.95 ± 3.68

**Table 2:** Molecular docking parameters for curcumin and imatinib with target proteins.

Protein (PDB ID)—Ligand Complex	Binding Energy (kcal/mol)	Dissociation Constant (μM)	Contacting Amino Acid Residues (H-Bonds)
I-Kappa-B-Alpha/NF-Kappa-B (PDB ID: 1NFI)—curcumin	−10.45	0.022	Gln 111
I-Kappa-B-Alpha/NF-Kappa-B (PDB ID: 1NFI)—imatinib	−10.67	0.015	Tyr 181, Lys 326
Akt1 (binding pocket of AMP-PNP) (PDB ID: 4EKK)—curcumin	−11.30	0.005	Lys 158, Glu 234, Asp 439
Akt1 (binding pocket of AMP-PNP) (PDB ID: 4EKK)—imatinib	−9.81	0.064	Lys 179, Asp 439
NF-kappaB inducing kinase (PDB ID: 4G3D)—curcumin	−10.12	0.038	Leu 445, Gly 446
NF-kappaB inducing kinase (PDB ID: 4G3D)—imatinib	−8.52	0.569	Gly 446
NF-kappaB inducing kinase (binding pocket) (PDB ID: 4G3D)—curcumin	−9.81	0.064	Glu 440, Cys 444, Gln 479
NF-kappaB inducing kinase (binding pocket) (PDB ID: 4G3D)—imatinib	−5.05	200.06	Gln 479, Phe 535
Protein kinase B unphosphorylated (PDB ID: 1GZO)—curcumin	−11.67	0.003	Thr 313, Gly 335
Protein kinase B unphosphorylated (PDB ID: 1GZO)—imatinib	−11.16	0.007	Trp 334
Phosphorylated p70S6K1 (binding pocket) (PDB ID: 3A62)—curcumin	−9.31	0.150	Lys 123, Glu 143
Phosphorylated p70S6K1 (binding pocket) (PDB ID: 3A62)—imatinib	−9.10	0.212	Leu 175

## Data Availability

Not applicable.

## Supplementary Materials

The following supporting information can be downloaded at: https://www.mdpi.com/article/10.3390/metabo13010058/s1, Figure S1: Binding mode of curcumin (orange) with I-Kappa-B-Alpha/NF-Kappa-B (PDB ID: 1NFI) as assessed by molecular docking study, Figure S2: Overlapping of curcumin (orange) and imatinib (blue) molecules at the same binding area in I-Kappa-B-Alpha/NF-Kappa-B after aligning complexes obtained by molecular docking study, Figure S3: Partial overlapping of curcumin (orange) and imatinib (blue) molecules at the similar binding area in NF-kappaB inducing kinase (PDB ID: 4G3D) after aligning complexes obtained by molecular docking study, Figure S4: Partial overlapping of curcumin (orange) and imatinib (blue) molecules at the similar binding area in unphosphorylated protein kinase B (PDB ID: 1GZO) after aligning complexes obtained by molecular docking study, Figure S5: Binding mode of curcumin (orange) with Akt1 (binding pocket of AMP-PNP) (PDB ID: 4EKK) as assessed by molecular docking study, Figure S6: Binding mode of curcumin (orange) with NF-kappaB inducing kinase (binding pocket) (PDB ID: 4G3D) as assessed by molecular docking study, Figure S7: Binding mode of curcumin (orange) with phosphorylated p70S6K1 (binding pocket) (PDB ID: 3A62) as assessed by molecular docking study, Table S1: Absorbance values (OD 450 nm/620 nm) from WST-8 analysis of LAMA84R cell line incubated with DMSO and RPMI control for 48 h. The data represent absorbance values from three independent experiments performed in triplicates.
