# Supplementary material for: Curcumin Decreases Viability and Inhibits Proliferation of Imatinib-Sensitive and Imatinib-Resistant Chronic Myeloid Leukemia Cell Lines
Source: Metabolites. 2022 Dec 30;13(1):58. doi: 10.3390/metabo13010058 (PMC9863870; doi:10.3390/metabo13010058)
Supplement: Supplementary file 1 [file metabolites-13-00058-s001.zip › metabolites-2028571-supplementary.pdf]

Supplementary Materials:

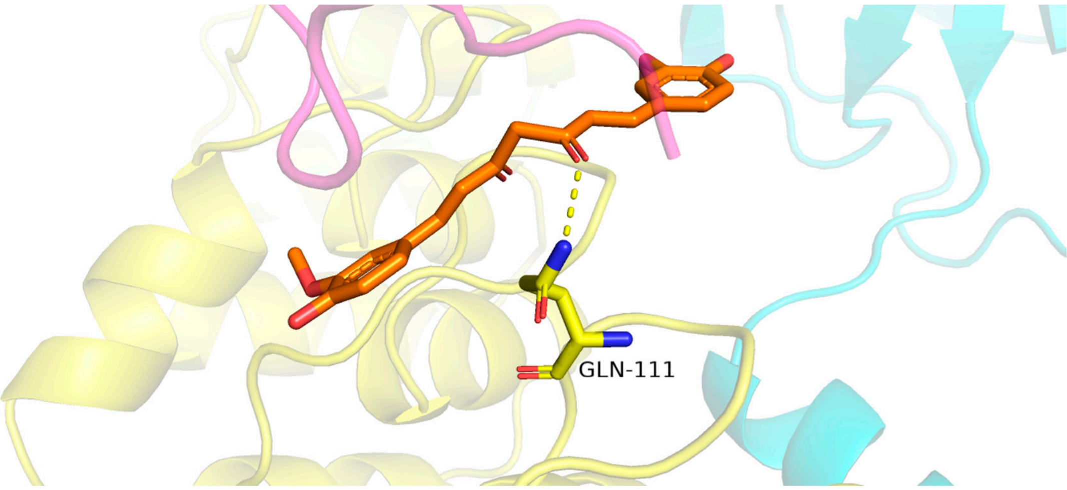

Figure S1. Binding mode of curcumin (orange) with I-Kappa-B-Alpha/NF-Kappa-B (PDB ID: 1NFI) as assessed by molecular docking study.

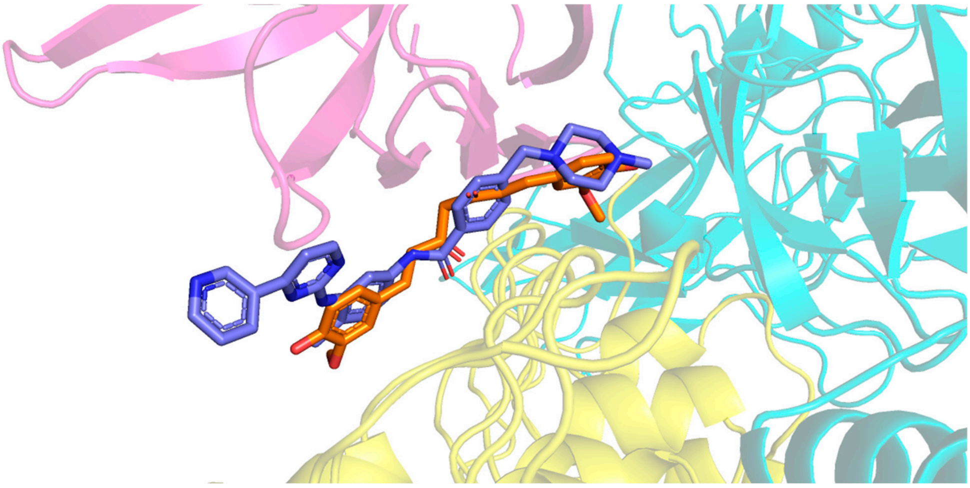

Figure S2. Overlapping of curcumin (orange) and imatinib (blue) molecules at the same binding area in I-Kappa-B-Alpha/NF-Kappa-B after aligning complexes obtained by molecular docking study.

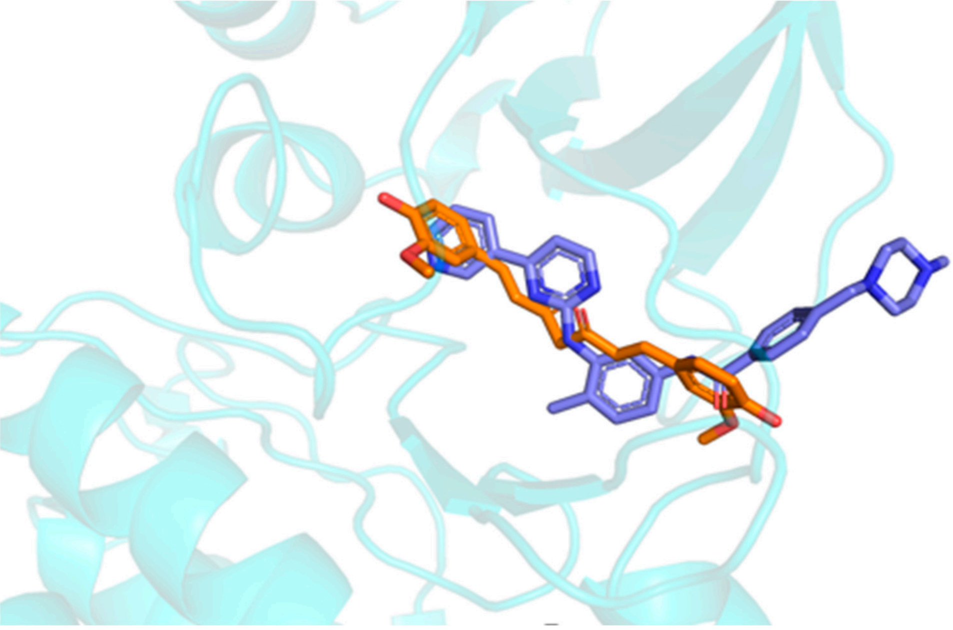

Figure S3. Partial overlapping of curcumin (orange) and imatinib (blue) molecules at the similar binding area in NF-kappaB inducing kinase (PDB ID: 4G3D) after aligning complexes obtained by molecular docking study.

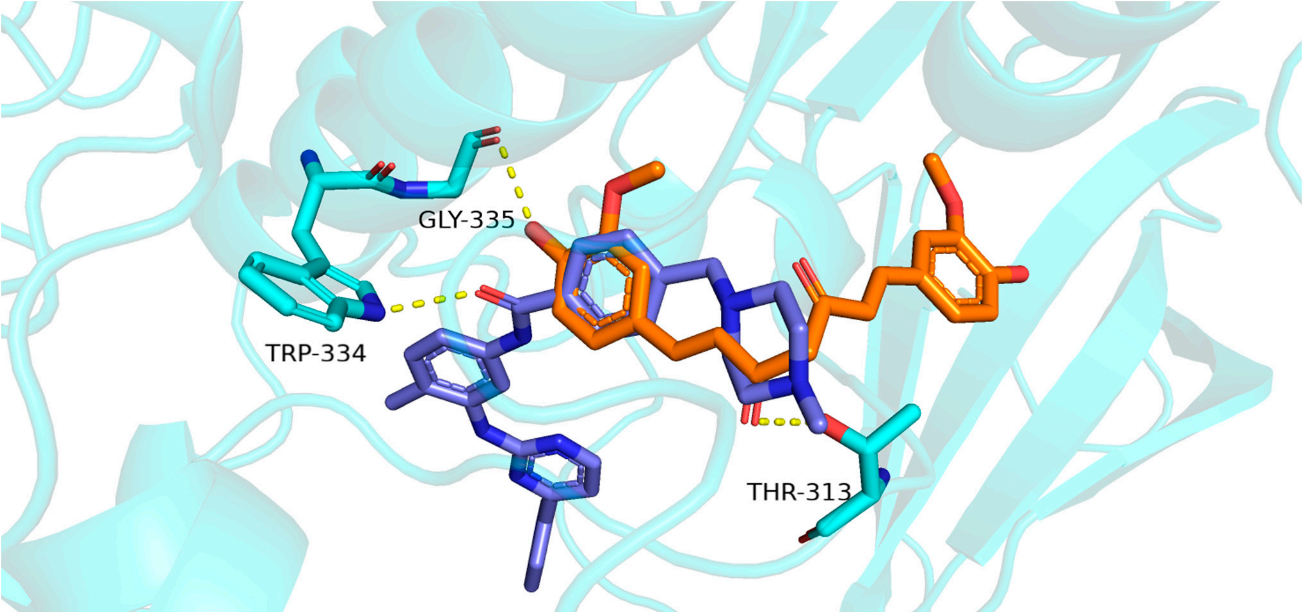

Figure S4. Partial overlapping of curcumin (orange) and imatinib (blue) molecules at the similar binding area in unphosphorylated protein kinase B (PDB ID: 1GZO) after aligning complexes obtained by molecular docking study.

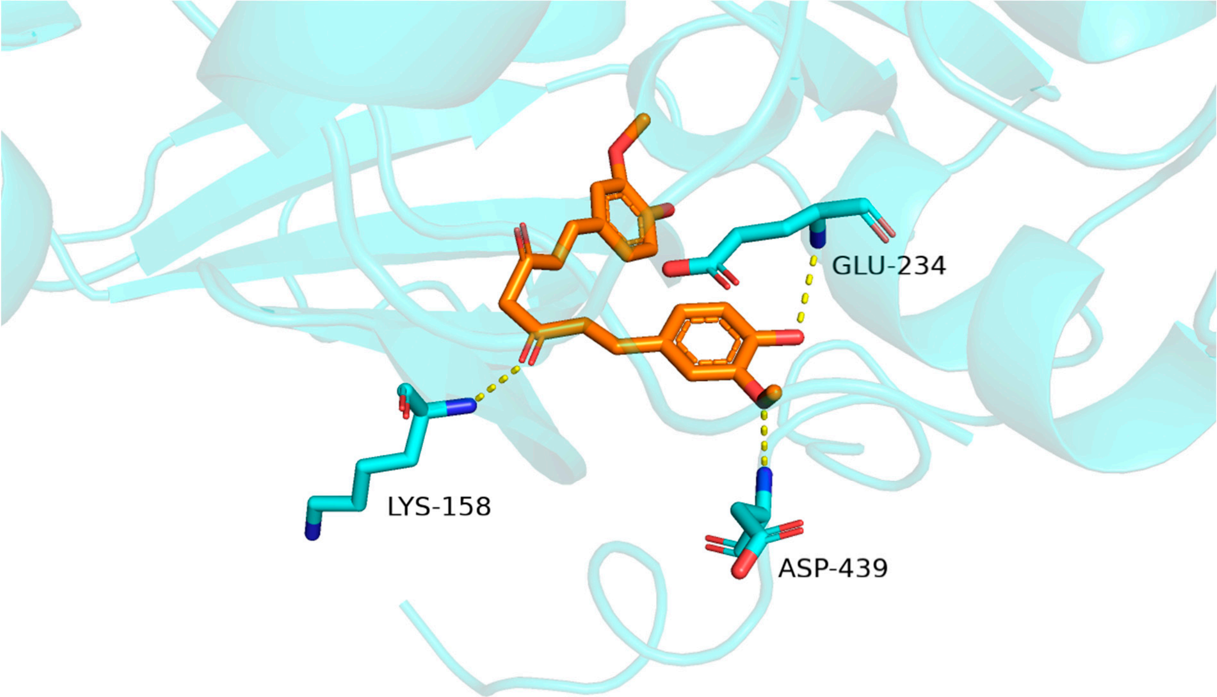

Figure S5. Binding mode of curcumin (orange) with Akt1 (binding pocket of AMP-PNP) (PDB ID: 4EKK) as assessed by molecular docking study.

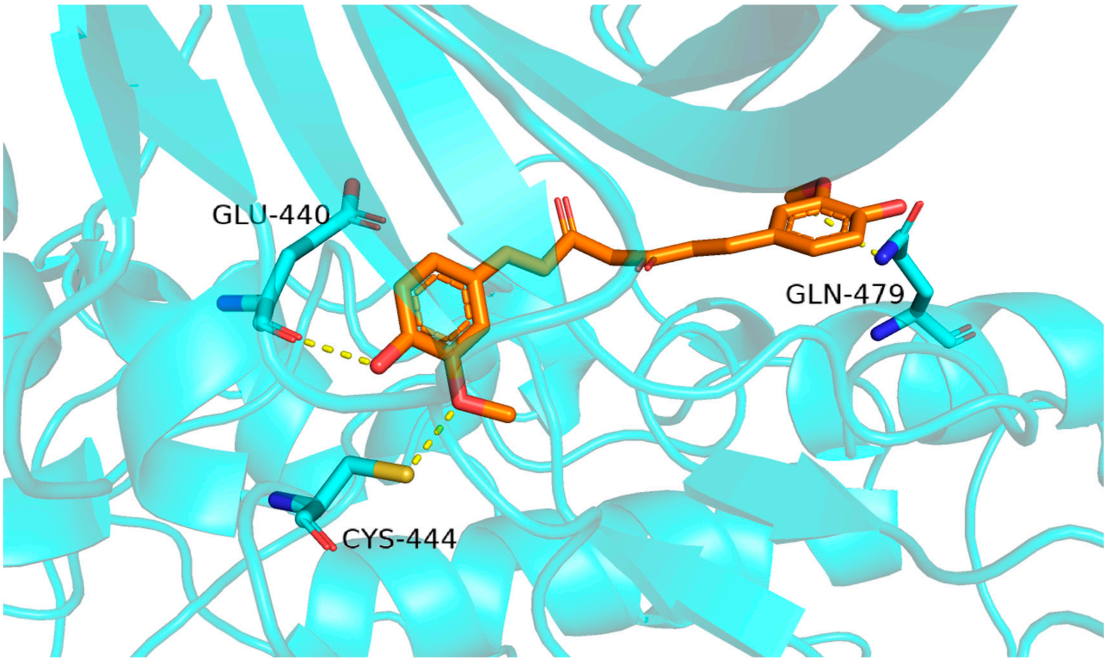

Figure S6. Binding mode of curcumin (orange) with NF-kappaB inducing kinase (binding pocket) (PDB ID: 4G3D) as assessed by molecular docking study.

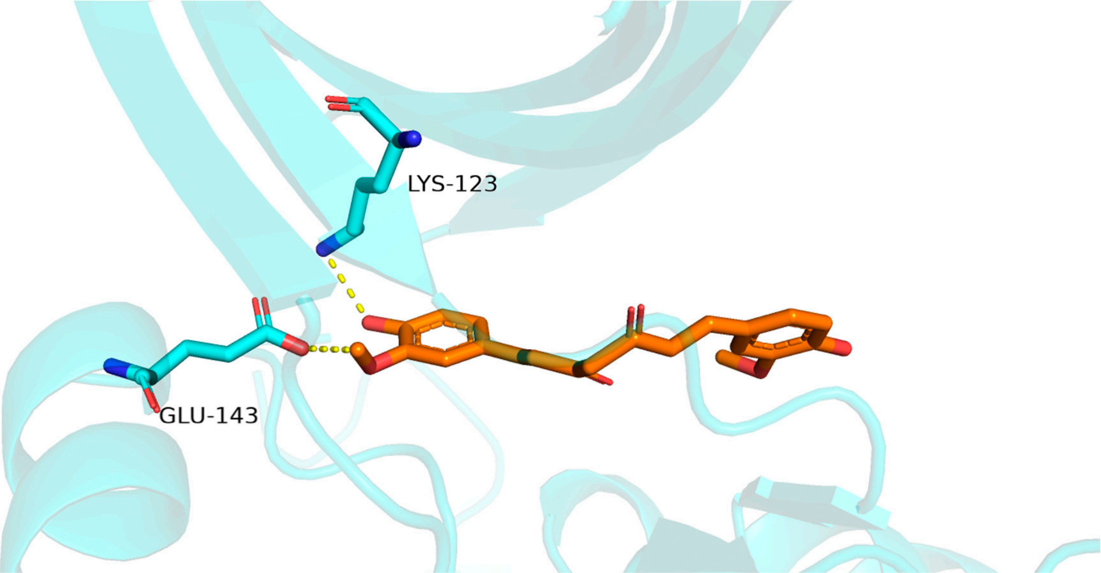

Figure S7. Binding mode of curcumin (orange) with phosphorylated p70S6K1 (binding pocket) (PDB ID: 3A62) as assessed by molecular docking study.

Supplementary Table S1. Absorbance values (OD 450 nm/620 nm) from WST-8 analysis of LAMA84R cell line incubated with DMSO and RPMI control for 48 hours. The data represent absorbance values from three independent experiments performed in triplicates.

	**Absorbance units**								
**Conditions**	Experiment 1			Experiment 2			Experiment 3		
DMSO Control	0.627	0.557	0.524	0.510	0.559	0.535	1.192	1.068	1.079
RPMI Control	0.562	0.556	0.572	0.544	0.565	0.555	1.197	1.067	1.086
